# The use of lumbar epidural injection of platelet lysate for treatment of radicular pain

**DOI:** 10.1186/s40634-017-0113-5

**Published:** 2017-11-25

**Authors:** Christopher Centeno, Jason Markle, Ehren Dodson, Ian Stemper, Matthew Hyzy, Christopher Williams, Michael Freeman

**Affiliations:** 1 Centeno-Schultz Clinic, Broomfield, CO 80021 USA; 2 Regenexx, LLC, Des Moines, IA 50321 USA; 3 CAPHRI School of Public Health and Primary Care, Maastricht University, Maastricht, Netherlands

**Keywords:** Platelet lysate, Lumbar, Radicular, Corticosteroids, Spine, Back pain, Regenerative, Steroids

## Abstract

**Background:**

Epidural steroid injections (ESI) are the most common pain management procedure performed in the US, however evidence of efficacy is limited. In addition, there is early evidence that the high dose of corticosteroids used can have systemic side effects. We describe the results of a case series evaluating the use of platelet lysate (PL) epidural injections for the treatment of lumbar radicular pain as an alternative to corticosteroids.

**Methods:**

Registry data was obtained for patients (*N* = 470) treated with PL epidural injections presenting with symptoms of lumbar radicular pain and MRI findings that were consistent with symptoms. Collected outcomes included numeric pain score (NPS), functional rating index (FRI), and a modified single assessment numeric evaluation (SANE) rating.

**Results:**

Patients treated with PL epidurals reported significantly lower (*p* < .0001) NPS and FRI change scores at all time points compared to baseline. Post-treatment FRI change score means exceeded the minimal clinically important difference beyond 1 month. Average modified SANE ratings showed 49.7% improvement at 24 months post-treatment. Twenty-nine (6.3%) patients reported mild adverse events related to treatment.

**Conclusion:**

Patients treated with PL epidurals reported significant improvements in pain, exceeded the minimal clinically important difference (MCID) for FRI, and reported subjective improvement through 2-year follow-up. PL may be a promising substitute for corticosteroid.

## Background

Lumbar epidural steroid injection (ESI) is a non-surgical treatment for radicular pain and low back pain (LBP). The procedure typically consists of an injection combining a corticosteroid anti-inflammatory with a local anesthetic into the epidural space below the level of the conus medullaris. ESIs are the most commonly performed pain management procedures for LBP and radiculopathy or radicular pain in the United States (Cohen 2011; Manchikanti 2004). From 1994 to 2001, the number of ESIs performed in the US increased by 629% (Deyo et al. 2009). Expenditure on ESI procedures has increased commensurately (Deyo et al. 2009; Manchikanti 2004). In 2012, the cost of ESIs exceeded $100 billion in the US (Wilkinson and Cohen 2012).

Despite the popularity of the procedure with pain management specialists and other clinicians, there is no clear evidence demonstrating the efficacy of ESI as a means of improving function, decreasing disability, or reducing spine surgery rates (Airaksinen et al. 2006; Armon et al. 2007; Deyo et al. 2009). Although some authors have reported mild to moderate short-term symptomatic improvement with ESI, long-term follow-up studies have not demonstrated a persisting benefit (Arden et al. 2005; Koes et al. 1995; Wilson-MacDonald et al. 2005). Indeed, a number of randomized placebo-controlled trials have concluded there is little difference in efficacy between ESI and placebo injections with saline (Deyo et al. 2009; Karppinen et al. 2001). It is also postulated the positive effects seen with ESI are more likely a result of the anesthetic component of the injection or the effect of diluting or displacing local inflammatory or noxious cytokines, rather than the anti-inflammatory action of the steroid (Manchikanti et al. 2015).

The therapeutic use of corticosteroids (used in high doses in ESI) is potentially associated with a multitude of adverse effects that can disrupt function of the endocrine, cardiovascular, musculoskeletal, gastrointestinal, dermatologic, metabolic, and nervous systems (Manchikanti 2002). Several authors have reported that repeated application of ESI can lead to significant bone loss and increased fracture risk in post-menopausal women (Kim and Hwang 2014; Mitra 2011; Bouvard et al. 2010). Other authors have demonstrated suppression of the hypothalamic-pituitary-adrenal axis persisting beyond 21 days post-ESI (Chon and Moon 2012). Reported adverse effects related to corticosteroid exposure include transient systolic blood pressure elevation and increased postprandial glucose levels that persisted for a longer time in diabetic versus non-diabetic patients (Kim and Hwang 2014; Younes et al. 2007).

Given the potential for negative health effects, as well as the equivocal evidence of efficacy for ESI, it is reasonable to seek out alternative non-operative treatments for lumbar radiculopathy or radicular pain. Regenerative therapies are a class of treatments, designed to replace, regenerate, or mitigate catabolism of damaged tissue. The therapies often utilize autologous blood products such as platelet rich plasma (PRP). PRP is the concentration of platelets obtained after isolation from the peripheral whole blood sample. PRP is used to promote healing and believed to act via the release of growth factors (GF) found inside platelets (Mautner and Kneer 2014). The inherent nature of autologous PRP has been shown in multiple studies to be safe, while the use of PRP for common orthopedic conditions such tendinopathies and osteoarthritis in peripheral joints has been promising (Sandrey 2014; Anitua et al. 2014). Published clinical evidence for PRP use in spinal conditions has been limited to lumbar spinal facet syndrome and intervertebral disc pathology (Wu et al. 2017; Tuakli-Wosornu et al. 2016; Kirchner and Anitua 2016; Akeda et al. 2017). Our own early clinical experience of injecting PRP into the intervertebral disc as part of a mesenchymal stem cell (MSC) procedure demonstrated no safety issues, which is consistent with reports from other authors (Levi et al. 2015; Centeno et al. 2011). Utilizing PRP for the treatment of radiculopathy has been limited to a small pilot study with *N* = 10, that found gradual improvements sustained through 3 months follow-up (Bhatia and Chopra 2016).

Platelet lysate (PL) is created by lysing platelets and removing the cell debris, resulting in a GF-rich injectate, devoid of other platelet material (Doucet et al. 2005). PRP and PL have both shown promise for clinical use in the treatment of orthopedic injuries over the past decade (Centeno 2014; Lopez-Vidriero et al. 2010; Randelli et al. 2014). Of note, there have been several studies documenting the beneficial effects of PRP for the treatment of peripheral neuropathy (e.g. carpal tunnel syndrome) and peripheral nerve regeneration after injury (Abbasipour-Dalivand et al. 2015; Anjayani et al. 2014; Elgazzar et al. 2008; Hibner et al. 2012; Kuffler et al. 2011a; Malahias et al. 2015). As an alternative to ESI for lumbar radiculopathy or radicular pain, PL may be preferable to PRP, as the former does not carry the potential for platelet adhesion and aggregation, which increases the risk of vascular occlusion (Wybier 2008). Given that several tragic cases of ischemic spinal cord lesions have been reported via particulate steroid occlusion of radicular arteries, scrutiny concerning the embolic nature of injectates is warranted (Pountos et al. 2016).

It has been widely reported that PL has the ability to promote the proliferation of various cell types including MSCs (Burnouf et al. 2016). Human serum (a significant component of PL) contains many different anti-inflammatory proteins including alpha-2-macroglobulin, interleukin receptor antagonist, and tissue inhibitor of metalloproteinase (Chen et al. 2014; Villeneuve et al. 2009). While the use of PL injected into the epidural space for the treatment of LBP and radicular pain has not been described previously in the literature, given its inherent anti-inflammatory and nerve repair potential, it may serve as an alternative to traditional ESI.

In the present study, we describe the analysis of patient tracking data from a prospective, multi-site registry. The data consist of outcomes and complications of the treatment of a consecutive sample of patients with lumbar radicular pain who underwent lumbar epidural PL injections and entered a registry. We believe this to be the first reported patient experience of the use of platelet products in the epidural space to treat lumbar radicular pain.

## Methods

Patients enrolled into a treatment registry designed to track patient safety and treatment outcomes. An Institutional Review Board (HHS OHRP #IRB00002637) approved the registry data protocol. All patients (or their guardians if they were under 18 years of age) enrolled in the registry underwent an informed consent process before entering the registry. Enrolled patients were prospectively followed using an electronic system, ClinCapture software (Clinovo Clinical Data Solutions, Sunnyvale, California) that generates automated pre- and post-treatment questionnaires for evaluation at 1, 3, 6, 12, 18, 24 months, and annually thereafter.

The registry protocol was as follows: patients were sent outcome and complications questionnaires. Up to five attempts were made at each time point to contact patients. If the patient failed to respond, that time point was lost to follow-up. Patients were exited from the registry at their request or if they elected for definitive surgery. For adverse events (AEs), broad tickler questions were asked to elicit a complaint. To enhance surveillance, all treating physicians were asked to report any complications related to the procedure. Any reported AEs were adjudicated by the treating physician and classified by relatedness and severity. For more details, please see the AE adjudication methodology already reported in prior publications (Centeno et al. 2011; Centeno et al. 2010; Centeno et al. 2016).

The data for the present investigation was culled from the patient registry database, representing 20 physicians at 13 outpatient interventional pain clinics treating patients with PL epidurals from November 2008 to August 2015. This sample included consecutive patients presenting with complaints of lumbar pain and radiating symptoms into their lower extremity. All patients were diagnosed with lumbar radicular pain based on history, physical exams, and MRI findings consistent with the diagnosis. Concurrent explanatory diagnoses included intervertebral disc derangements (e.g. herniation, protrusion, extrusion, etc.) and foraminal and/or central canal stenosis. The only inclusion criteria was that the patient have lumbar radicular pain in need of treatment, while exclusion criteria was based on medical conditions that would preclude an epidural injection route (e.g. coagulopathy, local infection at injection site, septicemia, pregnancy, and neurological disorders such as multiple sclerosis). Injection timing and frequency was based on clinical indication and was not controlled. Outcome measures obtained at each time point were a Numeric Pain Score (NPS), a modified Single Assessment Numeric Evaluation rating (SANE), and the Functional Rating Index (FRI).

NPS is a recognized way to quantify pain similar to a segmented version of the visual analogue scale, in which a whole number is selected to reflect the intensity of pain where 0 = no pain and 10 = worst possible pain (Katz and Melzack 1999; Hawker et al. 2011). The modified SANE rating asked patients what percent difference had they seen compared to their condition prior to the procedure from −100% worsened to 100% improved. This is a more rigorous version of the validated SANE metric which only allows responses from 0% to 100% (i.e. cannot determine if a patient reported a worse condition due to the procedure) (Williams et al. 1999; Shelbourne et al. 2012). The FRI instrument contains 10 items that assess pain and function focusing on activities of daily living that gauge patient disability. A higher score on FRI is indicative of more dysfunction with 0 representing functional independence with no limitations and 100 corresponding to severe disability (Feise and Michael Menke 2001).

Data for patients’ reported scores for each post-injection time point were analyzed for the three outcome measures. The differences between scores at pre- and post-treatment time points were calculated as change scores.

### Procedure description

Two weeks prior to undergoing the PL epidural procedure, patients were restricted from the use of corticosteroids and non-steroidal anti-inflammatory drugs. The rationale for the restriction is based on the concern for the inhibitory effect of such drugs on healing potential (Bondesen et al. 2004). On either the day of or day prior to the procedure, patients presented to the clinic for a venous blood draw, wherein approximately 60 mL of heparinized venous blood was collected. The heparinized venous blood was processed by hand in a sterile ISO-5 class biologic safety cabinet. No commercial automated systems designed to produce platelet rich plasma were used. To prepare the PL, whole blood was prepped via centrifugation at 200 g for 10 min to separate plasma from red blood cells. The resultant liquid lying above the concentrated solids (supernatant) was red blood cell / white blood cell poor and platelet rich. This 3-10 cm^3^ supernatant was extracted via pipette. Volume of supernatant varies based on patient’s hematocrit levels. All samples were then placed in a − 80° Celsius freezer for 5–10 min followed by thaw. This resulting blood product was then re-centrifuged to pellet any remaining platelet bodies, and the supernatant was extracted and sent to bedside for use. If the procedure was scheduled for the following day, the sample was placed in −20° freezer overnight and then re-thawed prior to use.

Accurate needle placement into the epidural space was accomplished utilizing C-arm fluoroscopy. Patients were treated with either a transforaminal or interlaminar epidural with the injection route determined by the examination and review of imaging. Detailed injection procedures have been previously described (Cohen 2011; Wilkinson and Cohen 2012). Once the needle was placed in the final target location and confirmed with fluoroscopy, Iodixanol (Visipaque, NDC# 0407–2223-06) radiographic contrast was injected to confirm flow into the epidural space. The final injectate consisted of PL 50% by volume, 4% lidocaine (NDC# 0409–4283-01) at 25% by volume, and compounded preservative free 100–200 ng/ml hydrocortisone (obtained via different compounding pharmacies) at 25% by volume. For transforaminal and interlaminar injections, 3-5 cm^3^ volume was injected. PL injectate was filtered at the time of injection with a 0.22-μm filter (Millex-GP, Millipore express PES membrane) to remove all remaining lysed platelet membrane debris. The nanogram amount of hydrocortisone used with PL is one million times less than what is commonly used for ESI injections and is used for its anti-inflammatory properties at levels similar to endogenous glucocorticoids (Jung et al. 2014).

Post-treatment, the patients were given instructions to participate in activity as tolerated. All patients were encouraged to participate in physical therapy, though this was not required nor controlled. A physical therapy prescription was provided at the patient’s request.

### Statistical analysis

Baseline characteristics were described using the mean and standard deviation for continuous variables. These included patient demographic information (age, body mass index (BMI) and gender), and self-reported scores (NPS, modified SANE Rating and FRI). Changes in the FRI and NPS scores were assessed using dependent two-group t-tests between the baseline and each post-treatment score. Subjects with missing scores were excluded from respective analyses. Differences between post-treatment time points were examined using a one-way analysis of variance (ANOVA) followed by post-hoc Tukey if warranted. All analyses were performed retrospectively utilizing R, a software environment for statistical computing and graphics, version 3.2.2.

## Results

**Fig. 1:**
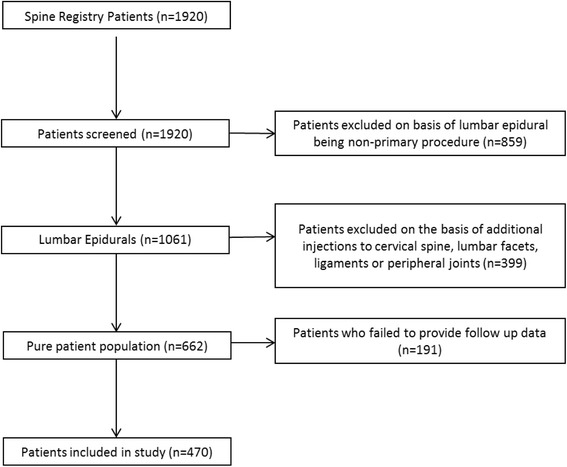
Study inclusion flow chart

The selection process for the patient population is shown in Fig. 1. Briefly, 1920 patients joined the registry tracking spine procedures. Of these, 859 received a spine procedure as a secondary injection to other diagnoses. Of the remaining 1061 patients, 399 received injections in the cervical spine, lumbar facets, lumbar ligaments, or peripheral joints, and were therefore removed. An additional 191 patients failed to provide follow-up data, resulting in final patient population of 470. This population consisted of 265 (56.4%) males and 205 (43.6%) females. Additional demographics are provided in Table 1. From the final sample, 325 were treated by 4 physicians at one site; the remaining 145 were treated at 11 other sites by 16 different physicians. Of the 470 patients, 273 (58%) had a single injection, 85 (18%) had two injections, 52 (11%) had three injections, and 60 (13%) patients had four injections, each at separate visits.

**Table 1:** Demographic information

Variable	*N*	Mean (SD)	Min	Max	
Age	470	53.6 (13.5)	16	91	
BMI	307	26.5 (4.5)	18.3	41.5	
Males	265 (56.4%)				
Females	205 (43.6%)				

Number of patients, mean and standard deviation included in each calculated variable with ranges for age and BMI

*BMI* body mass index, *N* number, *SD* standard deviation

### Adverse events

**Fig. 2:**
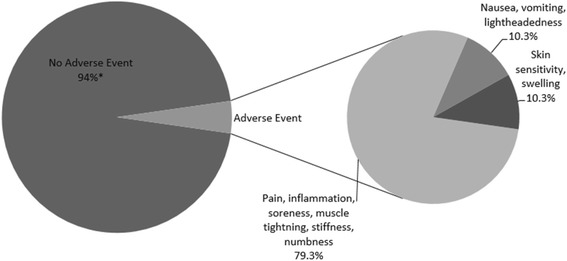
Adverse events reported. Adverse events (AE) related to the procedure were reported by 6.2% of patients. Of those 79.3% were categorized as pain related, 10.3% were dural puncture-related and 10.3% were skin reactions. *The percentage of patients without an AE related to the procedure

Of the 470 patients, 44 (9.4%) reported a complaint. Of those patients, 29 (6.2% of overall population) were adjudicated by the attending physician as an adverse event (AE) related to the procedure. Of those 29 patients, 4 reported AEs at multiple time points, with the majority being pain related. For example, 23 patients (79.3% of the AEs) reported pain related events, with a 2 patients reporting more than 1 event (pain (*N* = 18), inflammation (*N* = 1), soreness (*N* = 2), muscle tightening (*N* = 1), stiffness (N = 2) and/or numbness (*N* = 1). Other AEs were categorized as dural puncture related (i.e. nausea, vomiting with positional headaches and lightheadedness, *N* = 3) or skin reactions (redness or swelling, N = 3), which accounted for 20.7% of the total AEs. See Fig. 2. Of the three patients who reported symptoms consistent with dural puncture, two resolved with conservative care and the third resolved after receiving an autologous blood patch. No serious adverse events (infection, paralysis or neurologic deficit) were reported. Eleven patients (2.3%) reported that they elected for lumbar surgery: five within 5–7 months post-injection, two at 1-year, two at 18-months and two at 2-years. Over 93% of patients reported no AEs related to the procedure. All adverse events were self-limiting and resolved by 1–6 months.

### Numeric pain score

**Fig. 3:**
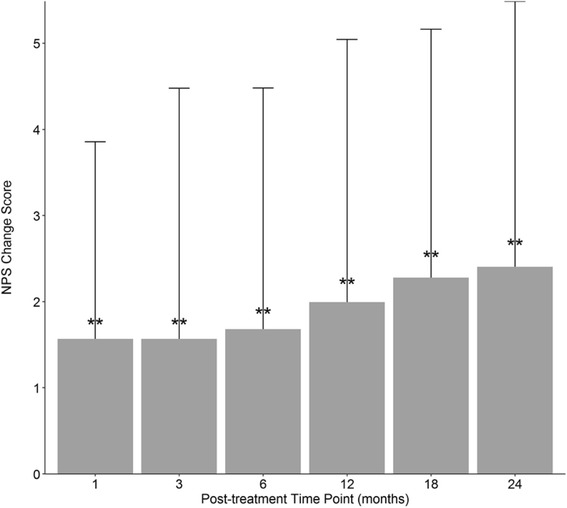
NPS average change scores. Numeric pain score (NPS) average change from baseline to post-treatment with standard deviation. Number of patients reporting at each time point: 1-month (*N* = 139); 3-month (*N* = 192); 6-month (*N* = 181); 12-month (*N* = 174); 18-month (*N* = 143); 24-month (*N* = 126). Statistical comparisons are to baseline. **p < .0001

Sixty-four percent of patients (*N* = 303) provided baseline NPS data, and those without baseline data were excluded from the respective analysis to limit missing data bias. Average baseline NPS was 5.1 (SD = 2.4). The change in scores from pre- to post-treatment was calculated for each patient at each time point. Change score averages ranged from 1.6–2.4, with each time point being statistically lower (*p* < .0001) than baseline (Fig. 3). Table 2 notes how NPS averages incrementally decreased from 1 through 24 months. An ANOVA showed NPS changed across post-treatment time points (*p* < .005). Post-hoc Tukey confirmed 18 month and 24 month NPS were significantly lower than 1 month (*p* < .05 and *p* < .01 respectively), as well as 24 month scores were significantly lower than 3 month scores (*p* < .05).

**Table 2:** Clinical outcomes for modified SANE, NPS and FRI

Time point	Modified SANE	NPS	FRI
Baseline	n/a	5.1 (303)	52.6 (239)
1-month	35.9 (128)	3.6 (139)	45.0 (111)
3-month	39.4 (219)	3.4 (192)	42.5 (144)
6-month	42.2 (239)	3.2 (181)	39.7 (146)
12-month	46.1 (245)	3.0 (174)	37.2 (136)
18-month	48.6 (210)	2.7 (143)	34.3 (114)
24-month	49.7 (198)	2.5 (126)	31.7 (100)

Mean (number of patients) of modified SANE rating, NPS and FRI at each time point

*FRI* Functional Rating Index, *NPS* Numeric Pain Score, *SANE* Single Assessment Numeric Evaluation

### Modified SANE rating

**Fig. 4:**
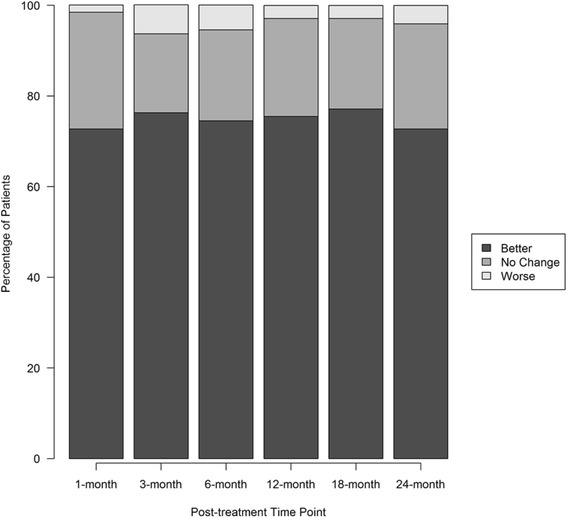
Modified SANE ratings tier plot. The percent of total patients at each post-treatment time point reporting feeling better (modified SANE > 0), no change (modified SANE = 0), or worse (modified SANE < 0). Patients reporting at each time point: 1- month (*N* = 128); 3-month (*N* = 211), 6-month (*N* = 216), 12-month (*N* = 203), 18-month (*N* = 153), 24-month (*N* = 129)

Of the 470 patients, 438 (93%) provided modified SANE rating responses. The patients’ last recorded responses averaged 42.4% improvement at an average of 16.6 months post-treatment. Modified SANE score means increased incrementally from 35.9% (SD = 34.2) at 1 month to 49.7% (SD = 42.1) at 24 months. A one-way ANOVA showed modified SANE scores differed significantly across time points (*p* < .005). Post-hoc Tukey showed significantly higher modified SANE scores between 1 month and both the 18 and 24 month post-treatment time points (*p* < .05). Scores trended toward a significant difference between the 3 month and 24 month time point (*p* = .07). Figure 4 displays the percentage of patients self-reporting improvement, no change, or a worse condition after receiving treatment. Improvement (modified SANE > 0) was noted in 72.7–77.1% of patients across the post-treatment time points. No change was reported in 17.4–25.8%, and a worse condition in 1.6–6.4% of patients. Detailed modified SANE ratings are characterized in Table 2.

### Functional rating index

**Fig. 5:**
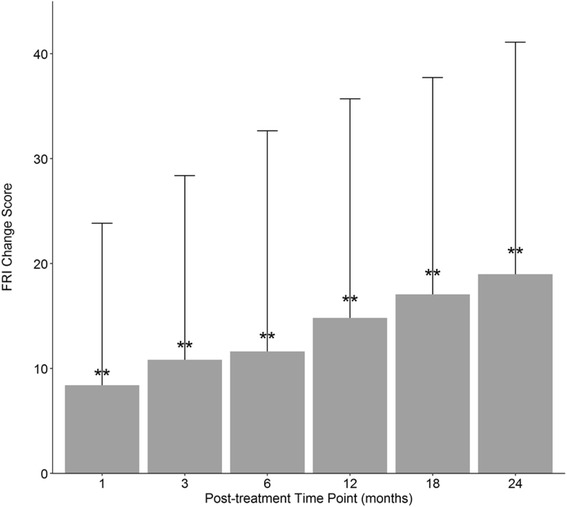
FRI average change scores. Functional rating index (FRI) averaged change in scores from baseline at each post-treatment time point with standard deviation. The number of patients reporting at each time point: 1-month (*N* = 111); 3-month (*N* = 144), 6-month (*N* = 146), 12-month (*N* = 136), 18-month (*N* = 114), 24-month (*N* = 100). **p < .0001

We obtained baseline functional rating index scores for 239 (51%) patients included in this analysis. Average baseline FRI score was 52.6 (SD = 20.6). The change in scores from pre- to post-treatment was calculated for each patient at each time point. The average change score at each time point was significantly different from baseline (*p* < .0001), with the average change score ranging from 8.4 to 19 (Fig. 5). The minimal clinically important difference (MCID) for FRI is 9 points, and was met or exceeded at all time points beyond 1 month (Childs and Piva 2005). Table 2 displays the incremental decrease in average FRI scores at each time point. An ANOVA showed scores differed between post-treatment time points (*p* < .0001). Post-hoc Tukey showed 12 month scores were mildly lower than 1 month scores (*p* = .052). Eighteen month scores were significantly lower than 1 month scores (*p* < .005) and 3 month scores (*p* < .05). Scores at 24 months were significantly lower than 1 month scores (*p* < .0005), 3 month scores (*p* < .005) and 6 month scores (*p* < .05).

## Discussion

Registry based data revealed PL epidural injections showed promise as an alternative treatment for lumbar radicular pain. Patients reported significantly less pain after treatment, maintained through 24 months. Self-reported modified SANE rating above baseline was demonstrated in the majority of patients with data (>72%) treated with PL at every time point, which was sustained through the 24 month follow-up period. Based on FRI change scores, function increased (indicated by scores decreasing) after treatment with PL, with average change scores exceeding the minimal clinically important difference for all time points beyond 1 month post-treatment. The significant difference in pain and function at later time points compared to scores at 1, 3, and even 6 months may suggest that a continued effect of the PL occurs over time.

Of the 470 patients, none reported any serious adverse events (life-threatening infections, hospitalizations, permanent neurologic deficit, or a complication that required significant care to mitigate) with the majority of AEs related to post-treatment pain that was self-limiting (79.3% of AEs). Other complications such as dural leaks were not more common than the expected rate for traditional ESIs (Epstein 2013). Rare adverse events included reports of lightheadedness, nausea, and vomiting. These may have resulted from a larger peripheral blood draw inducing a hypovolemic state in a minority of patients. In addition, eleven (<3%) of all patients opted for surgery after receiving treatment. In the current study, patients treated with PL epidurals improved their function and pain significantly from baseline at both short term (1, 3 and 6 months) and long term (12, 18 and 24 months) time points. The finding that patients reported clinically significant functional changes through 2 years is important to highlight. Previous ESI studies have shown a mild to moderate short-term benefit that was not sustained over long-term follow-ups, however, this comparison may or may not be applicable due to differences in study methodology (Arden et al. 2005; Koes et al. 1995; Wilson-MacDonald et al. 2005).

The properties of the growth factors and cytokines in PL may explain these findings, including enhancing nerve repair, improving blood supply, or reducing inflammation. These include transforming growth factor-beta (TGF-β), insulin-like growth factor-1 (IGF-1), vascular endothelial growth factor (VEGF), platelet-derived growth factors (PDGF), and others. Any or all of these acting individually or in conjunction may have accounted for the observed effects. For example, several GFs present in platelets have been identified as critical for nerve repair. PDGF stimulate the release of neurotrophic factors, which promote the regeneration of peripheral nerves (Monje et al. 2009). PDGF receptors are upregulated on axons and Schwan cells in injured nerves (Yamazaki et al. 2009). IGF-1 directly binds to IGF-1 receptors to stimulate the first step in fatty acid synthesis for re-myelination. The simultaneous application of PDGF and IGF-1 can induce more rapid regeneration than either of them individually (Oudega et al. 1997; Liang et al. 2007). VEGF induces angiogenesis, a critical step that precedes axon regeneration in animal models (Hoke et al. 2001). TGF-β can reactivate long-term denervated Schwann cells, triggering synthesis and release of neurotrophic factors, and thereby promote axonal regeneration (Johnson et al. 2008; Feng and Ko 2008). Kuffler and colleagues have shown in both animal models and case reports that the application of PRP can significantly improve peripheral nerve axon regeneration with gaps varying from 3 to 12 cm (Kuffler 2014; Kuffler et al. 2011b).

The procedure used in this study is quite different from more commonly used platelet rich plasma therapy, which was not used due to potential safety concerns regarding platelet aggregation. During epidurals, inadvertent vascular injections have been estimated at 8–12% (Epstein 2013). Coalescing platelets could lead to vascular occlusion and result in end-arterial capillary occlusion, similar to the proposed risks for particulate steroid injections (Wybier 2008). To eliminate this potential risk, PL was filtered through a 0.22 μm filter to eliminate the lysed platelet membranes prior to injection.

While the clinical use of PL compared to traditional PRP for orthopedic conditions has not been previously described, the effect of PL on human cells in vitro is well known. PL use for cell culture is described extensively in the literature and may provide some insight into its potential ability to stimulate local progenitor cells (Burnouf et al. 2016). Schallamoser et al. and several others have shown the benefits of culturing and expanding mesenchymal stromal cells (MSCs) in PL (Reinisch et al. 2007; Schallmoser et al. 2007). Overall, the clinical effectiveness of PL vs traditional PRP should be equivocal, the decision to use PL for epidural injection was based off the safety profile and a direct comparison would not be advisable given the potential risks of vascular occlusion with the use of PRP in the epidural space.

Limitations of the current study include lack of a control group and randomization, missing data, multiple components of the injectate, lack of strict post-treatment controls, and potential patient bias. This study did not include an untreated control group or randomization of subjects, hence placebo effects cannot be ruled out. The outcome data collected in the registry was missing various endpoints for patients, though multiple attempts were made to collect outcomes for each time point. Although this is an inherent problem with registry data, this platform allows us to prospectively monitor long-term outcomes in a large population of patients. In addition, the treatment injectate contained platelet lysate, local anesthetic, and a nanogram dose of corticosteroid, any of which may have produced the clinical effect seen, either alone or in combination. However, the volume of local anesthetic and corticosteroid is dramatically less than what has been compared for epidural therapeutic purposes, which leads us to believe that it is the PL producing the clinical outcomes in this sample of patients (Manchikanti et al. 2012). Post-treatment rehabilitation was not controlled, which could have led to differences in therapeutic effects. In addition, one cannot rule out potential bias introduced from patients paying for this therapy as part of their medical care.

## Conclusion

This is the first publication highlighting the clinical use of platelet lysate injected into the lumbar epidural space for the treatment of lumbar radicular pain. The analysis of the registry data shows promise for use of this therapy to reduce pain and improve function. We observed no additional risk beyond traditional steroid epidurals. While there is a need for randomized controlled trials to determine efficacy, these results highlight a potentially promising alternative to reduce the deleterious effects of high dose steroids on patients, as well as offer a possible non-surgical alternative for patients with lumbar radicular pain.

## Abbreviations

AE: Adverse events
ANOVA: Analysis of variance
BMI: Body mass index
ESI: Epidural steroid injection
FRI: Functional rating index
GF: Growth factors
IGF-1: Insulin-like growth factors-1
LBP: Low back pain
MCID: Minimal clinically important difference
MRI: Magnetic resonance imaging
MSC: mesenchymal stem cell
NPS: Numeric pain score
PDGF: Platelet derived growth factors
PL: Platelet lysate
PRP: Platelet rich plasma.
SANE: Single assessment numeric evaluation
TGF-β: Transforming growth-beta
VEGF: Vascular endothelial growth factors
